# Impact of Hypertension on Physical and Cognitive Performance Under Single- and Dual-Task Conditions in Older Adults

**DOI:** 10.3390/jcdd12100393

**Published:** 2025-10-04

**Authors:** Daniel Estévez-Caro, María Melo-Alonso, Miguel A. Hernández-Mocholí, Santos Villafaina, Francisco Javier Domínguez-Muñoz

**Affiliations:** 1 Physical Activity and Quality of Life Research Group (AFYCAV), Faculty of Sport Science, University of Extremadura, 10003 Cáceres, Spain; mmeloa@unex.es (M.M.-A.); mhmocholi@unex.es (M.A.H.-M.); fjdominguez@unex.es (F.J.D.-M.); 2 Facultad de Educación, Psicología y Ciencias del Deporte, Universidad de Huelva, 21071 Huelva, Spain

**Keywords:** balance, elderly people, exercise, gait analysis, motor skills, strength

## Abstract

Background: Up to 40% of people with hypertension (HTN) develop mild cognitive impairment and Alzheimer’s disease during their lifetime. This study aimed to compare physical and cognitive performance in older adults, classified as non-HTN or with HTN, under single-task (ST) and dual-task (DT) conditions. Methods: In total, 46 individuals (71 ± 5.96 years), divided equally into non-HTN and HTN groups, participated. Normality of the data was tested using the Shapiro–Wilk test. In this cross-sectional study, groups were compared using the Mann–Whitney U test applied to non-parametric variables and the independent samples *t*-test applied to parametric ones. Physical and cognitive functions were evaluated using the Short Physical Performance Battery (SPPB), HandGrip Strength (HGS), Timed Up and Go (TUG), and the L-Test, both in ST and DT conditions (with arithmetic tasks). Results: Significant differences were observed between groups in MoCA and the physical performance of SPPB, TUG, and L-Test under ST. In the DT condition, differences were found in the physical performance of TUG, L-test, and SPPB total score as well as in different components such as the 3 m walk and the Sit to Stand (STS). Regarding physical–cognitive interference, there was a statistically significant difference in the SPPB dual task cost between the HTN and non-HTN groups. Conclusions: Individuals with HTN exhibit impairments compared to non-HTN individuals in physical performance under DT conditions as well as in physical–cognitive interference. Static balance and HGS appear unaffected; however, differences are evident in gait (TUG and L-Test) and lower-limb strength (STS).

## 1. Introduction

Hypertension (HTN) is a generally chronic condition, characterized by a sustained increase in blood pressure [1], with reference values being 140 mmHg in systolic blood pressure and 90 mmHg in diastolic blood pressure [2]. It is one of the most common predictors of cardiovascular disease [3], affecting up to 1.4 billion adults (30–79 years) worldwide and 30% of people over the age of 20 [4,5]. Despite the large number of people who suffer from it, 40% are unaware of it, earning it the name ‘the silent killer’ [2,4,5]. Moreover, its prevalence generates costs of up to EUR 10.7 million per year [6].

There are numerous types of HTN, and according to their characteristics, they can be classified as primary or essential and secondary [3]. Primary HTN accounts for up to 95% of cases [7], with an idiopathic and multifactorial origin, where genetic predisposition, lifestyle, and age are among the most common risk factors [3]. On the other hand, secondary HTN makes up the remaining 5% [7], comprising known causes, such as diseases (mainly renal and endocrine) [3,8], or associations with specific conditions such as pregnancy [9].

Physical exercise is a key non-pharmacological element in reducing and/or preventing risk factors and symptoms of disease, including hypertension [10,11,12]. These benefits are obtained by the physiological adaptations that occur, such as the increase in maximal oxygen uptake (VO2 max) [11]. The most beneficial and rapid adaptations for HTN are generated by aerobic endurance training, such as High-Intensity Interval Training (HIIT) [11], and strength training, especially isometric strength [13,14].

Up to 40% of people with HTN develop mild cognitive impairment and Alzheimer’s disease during their lifetime [15]. This is an intermediate state between normal cognition and dementia, which is prevalent in older age, and its frequency increases with age [16] and can affect cerebrovascular structures and functions, directly damaging the brain over time [15]. In addition, it has also been observed that some drugs used to treat hypertension cause cognitive impairment (CI) [15,17]. It is important to detect cognition problems before they become more serious, to prevent and delay diseases such as dementia or CI [16]. Moreover, it is equally important that this strategy be accompanied by physical exercise to work on the prevention of different diseases, or in the case of the present investigation, the prevention and management of disease progression and the control of key health markers, such as aerobic fitness, in individuals with hypertension [11,16].

Certain tests to evaluate CI, such as the Montreal Cognitive Assessment (MoCA), have been used by different health professionals and scientists. However, this questionnaire does not assess the cost of performing a motor and cognitive task at the same time. A single task (ST) is one that is performed with a single attentional focus, and a dual task (DT) is one in which the user must perform two or more tasks at the same time [18]. By simultaneously involving physical and cognitive tasks, performance can be compared to that in a single physical task condition to determine whether CI exists [18,19]. However, few studies have explored the impact of DT on people with HTN. In this regard, research on CI has combined general DT with continuous walking, showing motor alterations in gait and increased activation of various brain regions [19,20].

Thus, the aim of the present study is to compare the physical and cognitive performance of two groups of elderly people (non-HTN and with HTN) in single- and dual-condition tests. We hypothesized that people with HTN will show lower performance in physical fitness tests involving gait, agility, and/or dynamic balance.

## 2. Materials and Methods

### 2.1. Design and Participants

The research follows a cross-sectional design, with a total sample of 46 subjects (4 men and 42 women). It is divided into the non-HTN group, made up of 23 non-HTN people, and the HTN group, made up of 23 people whose only disease is HTN.

The inclusion criteria for the selection of the sample were as follows: (a) being aged 55 years or older at the time of measurement, (b) being diagnosed with hypertension by their doctor, and (c) understanding the test protocols. The exclusion criteria were as follows: (a) suffering from any disease for which there is evidence of involvement in CI, and (b) suffering from any disease that conditions the performance of the tests according to the protocol.

Before starting the tests, each participant signed an informed consent form setting out all aspects of the research, approved by the Bioethics and Biosafety Commission of the University of Extremadura (approval code 37/2025).

### 2.2. Material and Instruments

Participants provided a series of personal data (date of birth (age), sex, educational level, marital status, and cohabitation). Questionnaires were administered to measure the level of physical activity and cognitive aspects, which were extracted thanks to the International Physical Activity Questionnaire in its short version and in Spanish (IPAQ-5) [21] and MoCA [22], respectively. Likewise, the measuring rod (ADE MZ10042, ADE Germany GmbH Hamburgo, Germany) was used to obtain height (m), and the bioimpedance meter (Omron Healthcare BF511, OMRON Healthcare, Kioto, Japón) was used to obtain weight (kg), fat, muscle and visceral percentages, and basal metabolism (Kcal).

The tape measure was used for the measurement of four perimeters (waist, hip, tricipital, and calf) (cm), and the chronojump (Chronojump-BoscoSystem, Barcelona, Spain) and a manual stopwatch were used for the duration of the tests (s). Finally, the chair used for the assessments was fixed, with a backrest prepared for the chronojump and without armrests.

Montreal Cognitive Assessment (MoCA). This test assesses different cognitive skills on a scale of 0–30, with results below 26 CI indicators [22]. The cognitive skills assessed are attention, concentration, executive functions (abstraction capacity), memory, language, visuoconstructive abilities, calculation, and orientation.

Short Physical Performance Battery (SPPB). This is a battery that assesses the functional capacity of the lower body by measuring balance, gait, and strength in different physical tests [23]. The results are shown on a scale of 0–12, dividing it into dependent (0–3), fragile (4–6), pre-fragile (7–9), and robust (10–12) [24].

HandGrip Strength (HGS). This is a test that measures the maximum isometric strength of a subject, using a hand-held dynamometer. The subject must stand upright with their arms stretched to hip height and apply strength, performing two repetitions with each hand [25]. The one used for the present study was the Takei TKK 5401 (Takei Scientific Instruments Co., Ltd., Tokyo, Japan), with analog grip and an adjustable handle, according to the size of each hand, with a range of 5–100 kg.

Timed Up and Go (TUG). This is a dynamic gait and balance test, where the subject starts from a chair and has to get up without using their hands, move to a cone 3 m away, go around it, and sit down again in the shortest possible time. Although it varies with age, if it exceeds 12 s, the risk of falling is high [26].

L-Test. This is an extension of the TUG, where functional ambulation is assessed [27]. The subject starts sitting on a chair, gets up and walks to a cone located 3 m away, turns 90° to the right at the far end, and walks to the next cone located 7 m away. Once there, they go around the cone and walk the same route until they are back in the chair. The estimated time for people who do not require walking aids should be less than 25 s [28].

### 2.3. Procedure

After collecting personal and anthropometric data and completing both questionnaires (IPAQ and MoCA), the cognitive test and physical tests were performed in single or dual condition. The tests have a fixed order, but both the type of condition of the physical tests and the arithmetic cognitive test were randomized.

The tasks that each subject had to complete during the assessment were as follows: (1) cognitive test: arithmetic-based counting test [29]; (2) motor task: SPPB, HGS, TUG, and L-Test; and (3) dual task: SPPB, HGS, TUG, and L-Test.

According to the different types of cognitive tasks defined by Tomas-Carus et al. [18] and the adaptations of Leon-Llamas et al. [30], the model chosen for the study was the arithmetic one, subtracting from 2 by 2, starting at a random odd number greater than 100. The duration was that of the test, except for the HGS where it was 5 s.

### 2.4. Statistical Analysis

The SPSS statistical package (version 30.0; SPSS, Inc., Chicago, IL, USA) was used for the overall data analysis. Additionally, the R statistical software (version 4.x; R Core Team, 2024) and the *pwr* package were used specifically to calculate statistical power.

To determine the normality of the sample, Shapiro–Wilk tests were performed. The independent samples T-test was performed for normal variables, and the Mann–Whitney U-test for non-normal variables, to observe the difference between the two groups (non-HTN group and HTN group), as well as to analyze the differences between ST and DT in both types. In addition, the dual task cost (DTC) was calculated for each type of DT. This is calculated as follows:

DTC = (outcome of the DT condition − outcome of the ST condition)/outcome of the ST condition(1)

Therefore, the Wilcoxon signed-rank test was also used to examine the differences between the DTC of the two types of DT.

For intra-group comparisons, the related-samples *t*-test was performed for parametric variables and the Wilcoxon signed-rank test for non-parametric variables.

The effect size was calculated using r for nonparametric variables and Cohen’s d for parametric variables [31]. Values of 0.81, 0.51, and 0.21 represent large, medium, and small effect sizes, respectively, according to Cohen’s classification [32]. The alpha level of significance (0.05) was adjusted according to the Benjamini–Hochberg procedure to avoid type I errors arising from multiple comparisons [33].

Post hoc statistical power was estimated for each comparison between the HTN and non-HTN groups, using the observed effect sizes (Cohen’s d or Hedges’ g, as appropriate) and a sample of 23 participants per group. Calculations were performed using the *pwr.t.test()* function of the *pwr* package in R [34], considering a bilateral test for independent samples and a significance level of α = 0.05. For effects calculated with the Hedges correction (g), their functional equivalence to Cohen’s d was assumed for the purposes of this analysis.

## 3. Results

The study was carried out by a total of forty-six people with a mean age of 71.87, although one of them was unable to complete the study due to medical reasons. Table 1 and Table 2 show the descriptive socio-demographic characteristics and percentages of the 46 subjects who were able to complete the study. In addition to general data, the different socio-demographic aspects are also shown by groups (non-HTN group and HTN group).

The questionnaires applied in the study (Table 1) show that there is homogeneity between groups in the EQ-5D-5L. However, both groups obtained values with a difference of more than 2 points (24.74 non-HTN and 22.39 HTN) in the MoCA, generating a mean of 23.57, a score that is at the low normal limit [35]. However, in the HTN group, their values are normal for people with their pathology [36]. MoCA is the only one of all the variables in Table 1 in which there are statistically significant differences (*p*-value = 035).

On the other hand, the anthropometric analysis of the sample (Table 1) presents a mean Body Mass Index (BMI) of 26.58 kg/m^2^, which represents a sample with overweight for its age [37], with a mean fat mass percentage of 38.01%, a lean mass of 25.49%, and a visceral mass of 10.09%. These values are always higher than the mean in the HTN group (27.17 BMI; 38.68% fat mass, 25.88% lean mass, and 10.82% visceral mass) and lower in the non-HTN group (25.99 BMI; 37.37% fat mass, 25.12% lean mass, and 9.40% visceral mass, respectively).

Table 2 shows how up to 42 (91.3%) of the participants are women, compared to 4 (8.7%) who are men. Both the employment situation and the IPAQ-5 and coexistence show quite similar results in both groups, with retired, low level, and living with spouse being the most frequent options, respectively.

Continuing with the academic level, higher education is the most common (39.1%), closely followed by primary education (34.8%). In the HTN group, these are much more common, at 43.5%, while in the non-HTN group, secondary education is in second position (30.4%), with higher education remaining in first position with a higher percentage than in the total sample (43.5%).

Finally, marital status is quite disparate between groups, although widowed is the most common option both in total (52.2%) and in the non-HTN and HTN groups (47.8% and 56.5%, respectively). However, it should be noted that in the non-HTN group up to 17.4% are single, compared to 0% in the HTN group.

Cognitive test performance (Table 3) did not show statistically significant differences between the HTN and non-HTN groups in the operations conducted (*p* = 0.497). Likewise, no statistically significant differences were observed in the error rate (*p* = 0.104) or in the number of hits (*p* = 0.353).

Table 4 and Table 5 show the results of the different physical condition tests, categorizing the data according to the groups and whether it is ST or DT, with the consequent items related to the DT such as hits, errors, operations, and DTC obtained during the test.

SPPB (ST). This showed significant differences between both groups (*p* = 0.018). The mean is slightly higher in the non-HTN group (11.87) than in the HTN group (11.35). Finally, the effect size is small to medium (0.349). In addition, it is worth noting the statistically significant difference in the STS (*p* = 0.018) with the same effect size (0.349).

SPPB (DT). This also showed statistically significant differences (*p* = 0.003), with the mean of the non-HTN group being higher than that of the HTN group (10.70 and 9.09, respectively). The effect size is medium to large (0.433). Also, the DTC was significantly different from the HTN and non-HTN group, showing more physical–cognitive interference in the HTN group.

Bipedestation and semitandem. Both of these show a perfect score by users (1 point). The hits are higher in the non-HTN group (4.26 in bipedestation and 4.13 in semitandem), compared to those in the HTN group (2.00 and 2.61, respectively). Both show statistically significant differences (*p* < 0.001 and *p* = 0.009, respectively). In turn, the effect size is medium in bipedestation (0.514) and in semitandem (0.383). The errors follow a similar line, with more errors in the HTN group (0.39 in bipedestation and 0.52 in semitandem) than in the non-HTN group (0.13 and 0.09, respectively). However, the differences are only significant in semitandem (*p* = 0.014), with a medium effect size (0.363). Regarding operations, the non-HTN group (4.39 and 4.22) performed more in bipedestation and semitandem than the HTN group (2.39 and 3.13). The differences are statistically significant (*p* = 0.001 and *p* = 0.048), with medium-to-large effect sizes (0.477 and 0.291, respectively). Finally, the DTC in both is 0.

Tandem. The result is perfect in the non-HTN group (2 points), but slightly lower in the HTN group (1.96). The difference is not statistically significant (*p* = 0.317). Hits and operations are higher in the non-HTN group (3.96 and 4.13) compared to the HTN group (2.83 and 3.52), with no statistically significant differences (*p* = 0.078 and *p* = 0.291, respectively). On the other hand, errors are more frequent in the HTN group (0.70) compared to the non-HTN group (0.17), with statistically significant differences (*p* = 0.005) and a medium effect size (0.418). Finally, DTC is close to 0 in both groups, with no significant differences (*p* = 0.317).

3 Meters. The group with the highest score is the non-HTN group, with 3.65 points, compared to 2.83 points for the HTN group. The differences are statistically significant (*p* = 0.008) with a medium-to-large effect size (0.393). Hits and operations are higher in the non-HTN group (1.87 and 2.00) than in the HTN group (1.30 and 1.70), with no statistically significant differences (*p* = 0.434 and *p* = 0.727). Errors are higher in the HTN group (0.39) than in the non-HTN group (0.13), with a marginally significant difference (*p* = 0.080) and a medium effect size (0.258). DTC did not show significant differences (*p* = 0.073).

Sit to Stand (STS). The score is higher in the non-HTN group (3.04) than in the HTN group (2.30), with statistically significant differences (*p* = 0.024) and a medium effect size (0.333). The rest of the parameters (hits, errors, operations, and DTC) are higher in the HTN group (6.00, 0.83, 6.83, and 0.50) than in the non-HTN group (4.96, 0.43, 5.39, and 0.35). These differences are not statistically significant (*p* = 0.116, *p* = 0.115, *p* = 0.403, and *p* = 0.660). Effect sizes are small in all cases.

HGS. The non-HTN group obtained the highest values with 49.70 (9.73) Kg/N in both hands: 25.03 (5.04) Kg/N in the right hand and 24.67 (4.99) Kg/N in the left hand. Furthermore, no statistically significant differences are observed in any of them (*p* > 0.05).

TUG and L-Test. Both tests show very similar results, with a lower time in the non-HTN group (6.53 s and 14.54 s) compared to the HTN group (7.65 s and 16.95 s). In both cases, the differences are statistically significant (*p* = 0.004 and *p* = 0.007), with medium effect sizes (0.428 and 0.395, respectively).

HGS Both Hands (DT). In line with the ST, the non-HTN group presented higher values (45.40 vs. 42.93 Kg/N), but the differences were not statistically significant (*p* = 0.356). For its part, the DTC was the same in both groups (−0.09), with no statistically significant differences (*p* = 0.860).

HGS Right (DT). Strength (23.13 Kg/N), hits (2.61), and operations (2.65) were greater in the non-HTN group compared to the HTN group (22.07 Kg/N, 2.09, and 2.30, respectively). None of these reached statistical significance (*p* = 0.525, *p* = 0.111, and *p* = 0.119). Errors were higher in the HTN group (0.22 vs. 0.04), but this difference was not statistically significant (*p* = 0.154). Similarly, the DTC was higher in the HTN group (−0.09 vs. −0.08), with no significant difference (*p* = 0.481). Effect sizes were small across variables.

HGS Left (DT). Strength was higher in the non-HTN group (22.27 Kg/N) compared to the HTN group (20.85 Kg/N), with no statistically significant difference (*p* = 0.367). However, hits and operations were significantly higher in the non-HTN group (2.72 and 2.80) than in the HTN group (1.70 and 1.78), with statistically significant differences (*p* = 0.028 and *p* = 0.020) and medium effect sizes (0.324 and 0.334, respectively). Errors and DTC did not differ significantly between groups.

TUG (DT). The time to perform the test was lower in the non-HTN group (7.28 s) compared to the HTN group (8.83 s), with statistically significant differences (*p* = 0.004) and a medium effect size (0.426). The remaining variables (hits, errors, operations, and DTC) showed no statistically significant differences (*p* > 0.05), with small effect sizes.

L-Test (DT). The test time was lower in the non-HTN group (16.42 s) than in the HTN group (19.47 s), with statistically significant differences (*p* = 0.001) and a large effect size (0.999). Hits and operations were also higher in the non-HTN group (6.65 and 7.30) compared to the HTN group (4.57 and 5.96). Only hits reached statistical significance (*p* = 0.038), with a medium-to-large effect size (0.628). Errors were higher in the HTN group (1.39) than in the non-HTN group (0.65), without reaching statistically significant differences (*p* = 0.059). Finally, DTC did not differ significantly between groups (*p* = 0.258).

At the intra-group level, the comparison between performance on the dual task and the single task in the SPPB, HGS, TUG, and L-Test tests shows a significant decrease in performance when two simultaneous tasks are applied (one motor and one cognitive).

## 4. Discussion

The aim of the present study was to compare the physical and cognitive performance of two groups of older people (non-HTN and with HTN) in single- and dual-condition tests. Although numerous investigators have studied HTN because of its high prevalence, few studies have met the objective proposed here. This study represents a pioneering effort to analyze whether such differences exist.

The SPPB is a set of tests that assess balance, gait, and strength, having evidenced in two of them, strength and gait, the affectation of DT in the results [38]. According to our results, we found significant differences exist between the two groups. This is consistent with previous results obtained in the research of Reddy et al. [39]. However, contrary to this research, the male/female ratio was favorable for men, which may slightly interfere with the comparison.

Previous studies, including those by Reddy et al. [39] (on subjects with HTN) or Einstad et al. [40] (on stroke survivors), support the statistically significant differences observed in the SPPB under dual-task conditions. Nevertheless, the SPPB is consistently evaluated as a composite score derived from all its subtests, without disaggregating the findings of each specific component. Consequently, the data related to the 3 m, STS, and the DTC of the STS test, despite showing notable statistical variations, lack direct support from prior studies conducted on comparable populations.

Despite these observations, a closer analysis of the aforementioned variables reveals significant differences (*p*-value = 0.008) and a large effect size (0.393) in the 3 m test under the DT condition. Also, in the STS, statistically significant differences were found in the ST and DT conditions. These findings could be due to damage in cerebrovascular structures and functions caused by HTN [15]. Among the affected functions could be the executive function, which governs cognitive processes such as planning, organization, and decision-making, relevant in dual-task performance. These processes collectively interfere with the STS test because, unlike other components of the SPPB, the STS test uniquely demands cognitive–motor coordination for correct execution.

Following on from the previous line, all the people in this study are robust; however, when DT was applied, the HTN group went from 11.35 in ST to 9.09, becoming virtually a prefrail group. This is something that did not happen in the non-HTN group, because despite reducing their results by a little more than one point (11.87 in ST vs. 10.70 in DT), they remained in the “robust” classification. This change was evidenced by the statistically significant differences found in the DTC between HTN and non-HTN for the SPPB. This research could support previous studies [15] where the relationship between HTN and CI is observed. This fact underscores the need for people with HTN to control the side effects of HTN, such as, in this case, CI. Thus, multicomponent training could be incorporated in this population, since previous studies have shown that, in less than two months, both physical and cognitive fitness are improved [41], or HIIT, which has also been shown to have superior improvements compared to other aerobic endurance training in people with CVD [11].

The HGS did not present statistically significant differences either in the individual tests performed in different conditions or comparing the results of both conditions. Nevertheless, previous studies found significant differences [38,42]. This could be due to the difference between the tests chosen for DT, since in Merchant et al. (2021) a memoristic task was used (different brain areas are involved in this task) [38]. In the case of arithmetic tasks, a larger area will be activated than in memory tasks, including the inferior parietal cortex or certain temporal regions, among others [43,44]. The areas that activate arithmetic tasks benefit from the cross-tasking of motor tests [45], which could justify the differences found with the study by Merchant et al. [38]. On the other hand, the results in operations and hits are like those obtained by Lau et al. [42].

Regarding the TUG test, statistically significant differences between groups were found in the ST condition, which strengthens the evidence of investigations that obtained very similar results [46,47] when studying the risk of falls in older people. On the other hand, in the L-Test, significant differences were found between groups in the ST condition, which align with the obtained results of Omana et al. [48] in people with lower limb prostheses or those of Frengopoulos et al. [49] in people with lower limb amputations. In the L-Test from the DT condition, there are also statistically significant differences between groups, coinciding with the investigations of Omana et al. [48] and Frengopoulos et al. [49], both in people with lower limb amputations, and those of Leon-Llamas et al. [50] in people with fibromyalgia. However, although the hits obtained during the test present statistically significant differences, this does not coincide with any of the studies, which may be due to the age difference in the subjects of the present investigation, as it stands at 71 years, compared to 62.9 in Omana et al. [48] and 60.36 (7.84) in Frengopoulos et al. [49]. When comparing both conditions in the L-Test, there are also statistically significant differences, which again supports previous studies such as Omana et al. [48], Frengopoulos et al. [49] or Leon-Llamas et al. [50], as well as others developed in subjects with fibromyalgia [51].

### 4.1. Limitations

This study has some limitations that should be acknowledged. The first is the small size of the sample, which was obtained only through the “El ejercicio te cuida” (Spain) program, due to the need for a medical report to validate the HTN. This small number did not allow for a second comparison to be made between the sexes, which would have provided more information on the relationship between HTN and CI. Secondly, although the results showed lower values in the group with HTN, as this was a cross-sectional study with a single intervention, it cannot be determined that this reduction was caused by HTN. Third is the scarcity of existing literature on the subject, which has conditioned the choice of physical fitness assessment tests, reducing them to those known through the program. Finally, blood pressure was not measured prior to testing, as it is usually monitored annually by physicians, and pharmacological treatment was assumed to be effective. However, detailed information on the medication used was not available due to the nature of the sample. These factors can be considered in the interpretation of the results. Also, although no comorbidities were diagnosed in the HNT group, the presence of undiagnosed conditions cannot be excluded.

### 4.2. Implications and Future Prospects

Firstly, the support of previous evidence on the existence of mild cognitive impairment in persons with HTN, together with the preliminary findings of the present study, suggests the potential value of implementing physical activity programs focused on those qualities that appear more likely to be affected by DT (lower body strength and gait). In addition, it opens the door to following the investigation of those qualities that are most affected in HTN by CI, which would facilitate the work of those professionals in charge of preventing and/or dealing with them. This future research could be conducted by performing dual tasks that are more similar to everyday life, such as talking on the phone.

## 5. Conclusions

People with HTN could have deficits in DT fitness tests as well as in physical–cognitive interference compared to non-HTN people. Static balance and HGS may not be affected; however, gait and dynamic balance (TUG and L-Test) and lower body strength (STS) could be impaired. These impairments observed under dual-task conditions may compromise functional autonomy in older adults with hypertension, potentially increasing their vulnerability to frailty.

## Figures and Tables

**Table 1:** Descriptive sociodemographic characteristics.

Variables	Total (N = 46) Mean (SD)	Non-HTN (N = 23) Mean (SD)	HTN (N = 23) Mean (SD)	*p*-Value
Age (years)	71.87 (5.96)	70.91 (5.85)	72.83 (6.04)	0.281
Questionnaires				
EQ-5D-5L	0.96 (0.06)	0.96 (0.05)	0.96 (0.06)	0.619
MoCA	23.57 (4.03)	24.74 (3.48)	22.39 (4.27)	0.035 *
Anthropometry				
BMI (Kg/m^2^)	26.58 (4.20)	25.99 (3.82)	27.17 (4.56)	0.195
Fat mass (%)	38.01 (6.65)	37.37 (5.29)	38.68 (7.90)	0.515
Lean mass (%)	25.49 (4.49)	25.12 (4.20)	25.88 (4.85)	0.725
Visceral mass (%)	10.09 (2.99)	9.40 (2.33)	10.82 (3.46)	0.096

Note: SD: standard deviation; MoCA: Montreal Cognitive Assessment; *: *p*-value < 0.05; BMI: Body Mass Index.

**Table 2:** Sociodemographic characteristics by percentages.

	Total (N = 46)	Non-HTN (N = 23)	HTN (N = 23)
Variable	N (%)	N (%)	N (%)
Sex			
Man	4 (8.7%)	1 (4.3%)	3 (13.0%)
Woman	42 (91.3%)	22 (95.7%)	19 (87.0%)
Studies			
Primary	16 (34.8%)	6 (26.1%)	10 (43.5%)
Secondary	12 (26.1%)	7 (30.4)	5 (21.7%)
Superiors	18 (39.1%)	10 (43.5%)	8 (34.8%)
Employment status			
Retired	43 (93.5%)	21 (91.3%)	22 (95.7%)
Employed	1 (2.2%)	1 (4.3%)	0 (0.0%)
Self-employed	1 (2.2%)	1 (4.3%)	0 (0.0%)
Unemployed	1 (2.2%)	0 (0.0%)	1 (4.3%)
Civil Status			
Single	4 (8.7%)	4 (17.4%)	0 (0.0%)
Married	15 (32.6%)	6 (26.1%)	9 (39.1%)
Divorced	3 (6.5%)	2 (8.7%)	1 (4.3%)
Widowed	24 (52.2%)	11 (47.8%)	13 (56.5%)
Cohabitation			
Alone	9 (19.6%)	3 (13.0%)	6 (26.1%)
Family	8 (17.4%)	6 (26.1%)	2 (8.7%)
Parents	2 (4.3%)	1 (4.3%)	1 (4.3%)
Children	2 (4.3%)	1 (4.3%)	1 (4.3%)
Spouse	25 (54.3%)	12 (52.2%)	13 (56.5%)
IPAQ			
Low	46 (100%)	23 (100%)	23 (100%)
Moderate	0 (0.0%)	0 (0.0%)	0 (0.0%)
High	0 (0.0%)	0 (0.0%)	0 (0.0%)

**Table 3:** Cognitive test performance.

Variables	Non-HTN (N = 23) Mean (SD)	HTN (N = 23) Mean (SD)	*p*-Value	Effect Size
P.C Hits	10.83 (6.24)	9.09 (6.32)	0.353	0.277
P.C Error	0.96 (1.29)	1.52 (1.41)	0.104	0.240
P.C Op	11.78 (5.39)	10.61 (6.21)	0.497	0.202

Note. SD: standard deviation; P.C Hits: cognitive test hits; P.C Error: cognitive test errors; P.C Op: cognitive test operations.

**Table 4:** Motor and cognitive performance in SPPB in single- and dual-task conditions.

Variables	Non-HTN (N = 23) Mean (SD)	HTN (N = 23) Mean (SD)	*p*-Value	Effect Size
SPPB ST	11.87 (0.34)	11.35 (0.94)	0.018 *	0.349
SPPB DT	10.70 (1.11)	9.09 (1.95)	0.003 *	0.433
DTC	−0.10 (0.12)	−0.85 (0.10)	0.005 *	0.414
Bipedestation				
Score ST	1.00 (0.00)	1.00 (0.00)	1.000	<0.01
Score DT	1.00 (0.00)	1.00 (0.00)	1.000	<0.01
Hits	4.26 (2.03)	2.00 (1.70)	<0.001 *	0.514
Errors	0.13 (0.34)	0.39 (0.72)	0.215	0.183
Operations	4.39 (1.91)	2.39 (1.80)	0.001 *	0.477
DTC	0.00 (0.00)	0.00 (0.00)	1.000	<0.01
Semitandem				
Score ST	1.00 (0.00)	1.00 (0.00)	1.000	<0.01
Score DT	1.00 (0.00)	1.00 (0.00)	1.000	<0.01
Hits	4.13 (2.11)	2.61 (2.11)	0.009 *	0.383
Errors	0.09 (0.29)	0.52 (0.73)	0.014 *	0.363
Operations	4.22 (2.04)	3.13 (2.05)	0.048 *	0.291
DTC	0.00 (0.00)	0.00 (0.00)	1.000	<0.01
Tandem				
Score ST	2.00 (0.00)	2.00 (0.00)	1.000	<0.01
Score DT	2.00 (0.00)	1.96 (0.21)	0.317	0.147
Hits	3.96 (1.87)	2.83 (2.35)	0.078	0.532
Errors	0.17 (0.39)	0.70 (0.70)	0.005 *	0.418
Operations	4.13 (1.71)	3.52 (2.13)	0.291	0.316
DTC	0.00 (0.00)	−0.01 (0.04)	0.317	0.147
3 m				
Score ST	4 (0.00)	3.96 (0.21)	0.317	0.147
Score DT	3.65 (0.65)	2.83 (1.15)	0.008 *	0.393
Hits	1.87 (1.69)	1.30 (0.93)	0.434	0.115
Errors	0.13 (0.34)	0.39 (0.58)	0.080	0.258
Operations	2.00 (1.73)	1.70 (0.88)	0.727	0.052
DTC	0.43 (0.34)	0.90 (1.16)	0.073	0.264
STS				
Score ST	3.87 (0.34)	3.39 (0.84)	0.018 *	0.349
Score DT	3.04 (0.88)	2.30 (1.15)	0.024 *	0.333
Hits	4.96 (2.25)	6.00 (10.32)	0.116	0.232
Errors	0.43 (0.99)	0.83 (1.03)	0.115	0.233
Operations	5.39 (2.04)	6.83 (10.54)	0.403	0.123
DTC	0.35 (0.30)	0.50 (0.59)	0.660	0.065

Note. SD: standard deviation; *: *p*-value < 0.05; DTC: dual task cost.

**Table 5:** Motor and cognitive performance in HGS, TUG, and L-Test in single and dual conditions.

Variables	Non-HTN (N = 23) Mean (SD)	HTN (N = 23) Mean (SD)	*p*-Value	Effect Size
**Single Task**				
HGS				
Both hands (kg/N)	49.70 (9.73)	47.18 (9.58)	0.381	0.261
Right hand (kg/N)	25.03 (5.04)	24.01 (4.97)	0.492	0.204
Left hand (kg/N)	24.67 (4.99)	23.17 (4.93)	0.311	0.302
Other tests				
TUG (s)	6.53 (1.08)	7.65 (1.46)	0.004 *	0.428
L-Test (s)	14.54 (3.06)	16.95 (2.46)	0.007 *	0.395
Dual Task				
HGS both hands				
Score (Kg/N)	45.40 (10.61)	42.93 (10.21)	0.356	0.136
DTC	−0.09 (0.09)	−0.09 (0.09)	0.860	0.026
HGS right hand				
Score (Kg/N)	23.13 (5.61)	22.07 (5.61)	0.525	0.189
Hits	2.61 (1.12)	2.09 (1.56)	0.111	0.235
Errors	0.04 (0.21)	0.22 (0.52)	0.154	0.210
Operations	2.65 (1.07)	2.30 (1.66)	0.119	0.230
DTC	−0.08 (0.12)	−0.09 (0.10)	0.481	0.104
HGS left hand				
Score (Kg/N)	22.27 (5.38)	20.85 (5.11)	0.367	0.271
Hits	2.72 (1.50)	1.70 (1.55)	0.028 *	0.324
Errors	0.09 (0.29)	0.09 (0.29)	1.000	<0.001
Operations	2.80 (1.36)	1.78 (1.48)	0.020 *	0.334
DTC	−0.10 (0.10)	−0.10 (0.11)	0.886	0.021
TUG				
Score (s)	7.28 (1.96)	8.83 (2.14)	0.004 *	0.426
Hits	3.00 (1.60)	3.13 (2.53)	0.779	0.041
Errors	0.57 (0.79)	0.48 (0.79)	0.672	0.062
Operations	3.57 (1.38)	3.61 (2.41)	0.686	0.060
DTC	0.10 (0.15)	0.15 (0.19)	0.930	0.013
L-Test				
Score (s)	16.42 (2.41)	19.47 (3.58)	0.001 *	0.999
Hits	6.65 (3.35)	4.57 (3.27)	0.038 *	0.628
Errors	0.65 (0.98)	1.39 (1.37)	0.059	0.279
Operations	7.30 (2.99)	5.96 (2.72)	0.117	0.469
DTC	0.23 (0.61)	0.15 (0.14)	0.258	0.117

Note: SD: standard deviation; HGS: Manual Prehensile Strength; TUG: Timed Up and Go; *: *p*-value < 0.05; DTC: dual task cost.

## Data Availability

The data presented in this study are available on request from the corresponding author due to (The data will not be shown publicly, as patients will give their consent for the information to be kept confidential.).

## Abbreviations

The following abbreviations are used in this manuscript:

BMI	Body Mass Index
CI	Cognitive impairment
DT	Dual Task
HGS	HandGrip Strength
HIIT	High-Intensity Interval Training
HTN	Hypertension
IPAQ	International Physical Activity Questionary
MoCA	Montreal Cognitive Assessment
SPPB	Short Physical Performance Battery
SD	Standard Deviation
ST	Single Task
STS	Sit to Stand
TUG	Timed Up and Go
